# Interactive training with a novel simulation model for upper gastrointestinal endoscopic hemostasis improves trainee technique and confidence

**DOI:** 10.1055/a-2248-5110

**Published:** 2024-02-28

**Authors:** Takeshi Kanno, Yutaro Arata, Eric Greenwald, Paul Moayyedi, Suguo Suzuki, Yutaka Hatayama, Masahiro Saito, Xiaoyi Jin, Waku Hatta, Kaname Uno, Naoki Asano, Akira Imatani, Yutaka Kagaya, Tomoyuki Koike, Atsushi Masamune

**Affiliations:** 1 38047 Division of Gastroenterology, Tohoku University Graduate School of Medicine, Sendai, Japan; 2 12838 R & D Division of Career Education for Medical Professionals, Medical Education Center, Jichi Medical University, Shimotsuke, Japan; 3 73819 Graduate Medical Education Center, Tohoku University Hospital, Sendai, Japan; 4 62703 Division of Gastroenterology, McMaster University Faculty of Health Sciences, Hamilton, Canada; 5 13090 Faculty of Medical Science and Welfare, Tohoku Bunka Gakuen University, Sendai, Japan

**Keywords:** Non-variceal bleeding, Endoscopy Upper GI Tract, Ulcers (peptic and other), Quality and logistical aspects, Training

## Abstract

**Background and study aims** Endoscopic hemostasis is a life-saving procedure for gastrointestinal bleeding; however, training for it is often performed on real patients and during urgent situations that put patients at risk. Reports of simulation-based training models for endoscopic hemostasis are scarce. Herein, we developed a novel simulator called “Medical Rising STAR-Ulcer type” to practice endoscopic hemostasis with hemoclips and coagulation graspers. This study aimed to evaluate the reproducibility of the clinical difficulty of this model and the effectiveness of simulation-based training for clipping hemostasis.

**Patients and methods** This was a prospective educational study. Fifty gastroenterology residents from Japan and Canada were recruited to participate in a simulation-based training program. The primary outcome was the success rate for clipping hemostasis. We measured differences in trainee subjective assessment scores and evaluated the co-occurrence network based on comments after training.

**Results** The hemostasis success rate of the trainees significantly increased after instruction (64% vs. 86%, *P* < 0.05). The success rate for ulcers in the upper body of the stomach (59%), a high-difficulty site, was significantly lower than that for ulcers in the antrum, even after feedback and instruction. Trainee self-perceived proficiency and confidence significantly improved after simulation-based training ( *P* < 0.05). Co-occurrence network analysis showed that trainees valued a structured learning approach, acknowledged simulator limitations, and recognized the need for continuous skill refinement.

**Conclusions** Our study demonstrates the potential of our simulation-based training model as a valuable tool for improving technical skills and confidence in trainees learning to perform endoscopic hemostasis.

## Introduction

Endoscopic hemostasis for gastrointestinal bleeding is a life-saving procedure in urgent situations. In addition, it is a fundamental technique for treating bleeding complications of more advanced endoscopic treatments, such as endoscopic submucosal dissection and endoscopic mucosal resection (EMR), and should be learned by all endoscopy practitioners 1 . A systematic review of 75 studies reported that local injection therapy with epinephrine alone was insufficient for definitive hemostasis and was inferior to hemostasis with a hemoclip or coagulation grasper 2 . To achieve reliable endoscopic hemostasis, it is crucial to have an appropriate field of view based on stable endoscopic manipulation, proper control of devices such as hemoclips and hemostatic graspers, and coordination with surrounding doctors and nurses 2 . However, in most training programs, procedures are currently performed on real patients in urgent situations as on-the-job training. These stressful environments are not ideal for trainees to learn complex skills, and endoscopic techniques performed by unskilled trainees may put patients at risk. Furthermore, hemostatic procedures vary in difficulty depending on the bleeding site 3 , making it difficult to create a reproducible, standardized learning environment for trainees. Gastrointestinal bleeding remains a critical emergency with an approximately 10% mortality rate 4 , even though endoscopic procedures have been widely used for decades.

Simulation-based training (SBT) is a reproducible learning method performed in a calm environment, and there have been reports of SBT contributing to increased knowledge and improved skills, particularly in laparoscopic training 5 6 7 . The importance of SBT may apply to invasive procedures in general, as even reports from >1,800 surgeon training programs indicated limited clinical experience with major procedures in actual patients 8 . In the context of endoscopic training, SBT is effective for procedures such as the insertion of an esophagogastroduodenoscope/sigmoidoscope and ultrasound endoscopic imaging 9 10 11 . However, SBT for invasive endoscopic procedures has not been established properly, with only limited reports and evidence 12 . For example, an explanted porcine stomach model for endoscopic hemostasis training has been reported 13 ; however, such a model cannot be easily prepared and practiced repeatedly in regular clinical institutions or medical schools. When explanted organs are used as simulators, specialized endoscopes for the organ should be prepared, and concerns regarding the ethics of animal slaughter and hygiene issues, such as infection control, exist. Therefore, in collaboration with the Japanese industrial company Denka, we developed a novel simulator “Medical Rising STAR-Ulcer type” to practice endoscopic hemostasis with a hemoclip or coagulation grasper 13 . This study aimed to evaluate the reproducibility of the clinical difficulty of this model and the effectiveness of SBT with the model for clipping hemostasis.

## Patients and methods

### Study design and participants

This prospective educational study recruited 50 gastroenterology residents from tertiary referral university hospitals in Japan and Canada between February 2019 and August 2020. Only gastroenterology trainees were considered for inclusion. Medical students, junior residents, and trainees who did not consent were excluded from the study. The study protocol was approved by the Ethics Committee of Tohoku University (no. 2018–1-780). The study was conducted in accordance with the Declaration of Helsinki and Good Practice Guidelines and was registered with the UMIN Clinical Trials Registry (registration number UMIN000035735).

### Endoscopic hemostasis training program and evaluation of its effectiveness

Bleeding ulcers can be attached anywhere on the upper gastrointestinal luminal model. This simulator can be a valuable tool for practicing endoscopic hemostasis with real endoscopes and hemoclips, thereby improving the technical skills and confidence of trainees.Video 1

**Fig. 1:**
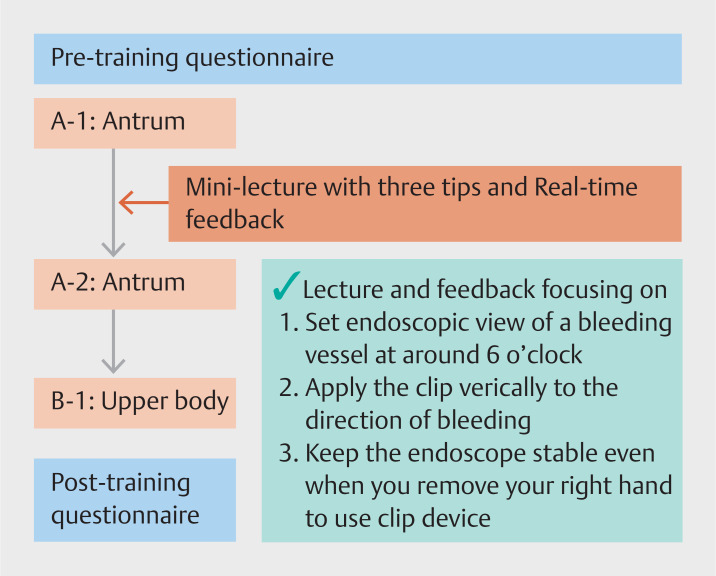
The endoscopic hemostasis training program flow using the “Medical Rising STAR-Ulcer type” model.

**Fig. 2:**
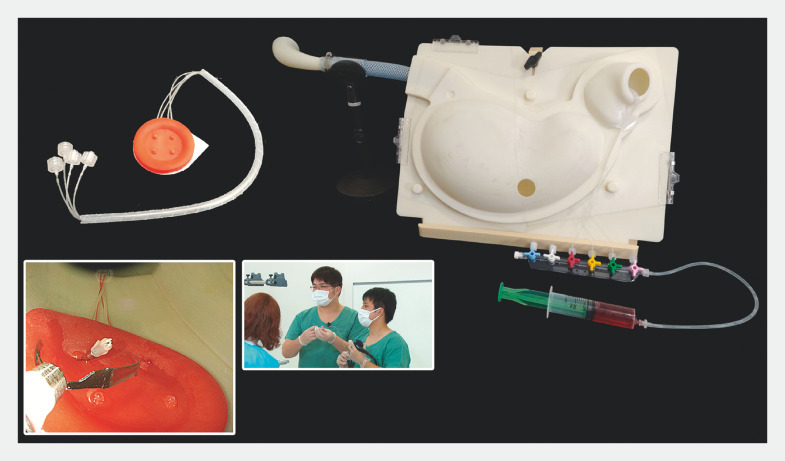
The appearance of the simulator model. The ulcer model can reproduce pulsatile bleeding from selected vessels that can be affixed anywhere in the stomach and duodenal lumen (upper left). Endoscopic image of simulated bleeding (bottom left). The lumen model from the pharynx to the duodenal portion: simplified from human 3D data and waterproof and impact-resistant (right).

Fig. 1 shows the flow of the training program. After providing informed consent, trainees completed questionnaires before and after training, which assessed their confidence in performing hemostatic procedures and the effectiveness of the training (Supplementary Fig. 1 and Supplementary Fig. 2). We created a mechanical simulator for endoscopic hemostasis (Medical Rising STAR -Ulcer type) 14 . Briefly, the ulcer model made of an elastomer resembling human mucosal elasticity could reproduce spurting bleeding from selected artificial vessels, and ulcers could be installed anywhere in the stomach lumen, which was designed based on human data ( Video 1 , Fig. 2 ). For this program, we used two bleeding gastrointestinal ulcers for each trainee: one placed on the greater curvature in the antrum of the stomach and one on the upper body of the posterior wall of the stomach. At least two expert endoscopists supported each trainee during the training. First, trainees attempted endoscopic hemostasis of the first vessel (A-1) of the antral ulcer without any feedback. Trainees could try up to three clips for a maximum of 15 minutes for each vessel. Time measurement started when the trainee recognized the ulcer lesion on the monitor. After completing the first trial without technical instruction, the expert endoscopists provided feedback and tips to the trainees, including tips regarding maintaining an appropriate view and distance from the ulcer, stabilizing the endoscope in position, and how to grasp an exposed vessel with a hemostatic clip.

The trainees then attempted hemostasis of the second vessel (A-2) at the same antral ulcer. After completing training in the antrum, they moved to a more technically challenging location: the upper body of the posterior wall of the stomach (B-1). In this simulator-based educational program, each trainee was individually instructed by two endoscopic experts. This approach was designed to prevent any potential biases or interactions that might arise from instructing multiple learners simultaneously. For the training, GIF H260 (Olympus Corp., Tokyo, Japan) and EG-2990K (Pentax Medical, Hoya Corp., Tokyo, Japan) scopes and HX-610–135 (Olympus Corp., Tokyo, Japan) hemoclips were used.

### Outcome measures

We aimed to perform a comprehensive evaluation of the efficacy including both objective measures of skill improvement and subjective measures of confidence enhancement. Our primary outcome was to evaluate the efficacy of the training program using our novel simulation model. To assess this, we compared the success rates of hemostasis for antral ulcer bleeding before (A-1) and after (A-2) instructions. Success was achieved when proper vessel grasping and hemostasis were accomplished within three clips and 15 minutes per vessel. Failure was defined as the inability to achieve hemostasis with three clips or if the procedure time exceeded 15 minutes per vessel. We also compared trainee self-confidence and subjective evaluation of their skills using a Visual Analog Scale (VAS; range of values: 0–100) before and after training.

The secondary outcome was to identify factors related to the difficulty of upper gastrointestinal endoscopic hemostasis and to evaluate how well this model reproduces the challenges encountered in clinical settings. To evaluate this, we compared the success rates of hemostasis for antral ulcers after instruction (A-2) and ulcers in the posterior wall of the upper body of the stomach (B-1), thus assessing the reproducibility of site-dependent difficulty within the model.

In addition, we measured each procedure time. Given that some cases were judged as failures because the procedure time reached 15 minutes, the procedure time was compared only for successful cases.

Finally, to clarify the overall impression and next target of the training program, we created a text-mining figure of the co-occurrence network with a Jaccard index > 0.1 based on free comments after the training course. The co-occurrence network allows visualization of how words are connected in sentences 15 16 and the Jaccard index is a statistical measure for calculating the similarity and diversity of words in sentences 17 .

### Statistical analysis

The results were analyzed on an intention-to-treat basis. Regarding basic characteristics and clinical information, continuous variables are presented as means and standard deviations, and dichotomous variables are presented as counts and percentages. Statistical differences between groups were analyzed using the chi-square test for categorical variables and the Mann-Whitney U test for continuous variables. The Wilcoxon signed-rank test was used to compare VAS scores for each question before and after training. *P* < 0.05 was considered statistically significant. Analyses were performed using StatsDirect statistical software, version 3.3.5. The co-occurrence network with a Jaccard index > 0.1 was made using Python.

For the analysis of trainee self-confidence and the subjective evaluation of their skills, we referred to an effect size of 0.5 based on simulator studies in non-endoscopic fields 18 . Using an α error of 0.05 and power of 0.8 for the Wilcoxon signed-rank test, we calculated the required sample size using G*Power 3.1 software, resulting in a total sample size of 28 individuals. Because this model is a novel endoscopic procedure simulator for gastrointestinal bleeding, we aimed for 50 participants to ensure sufficient statistical power.

## Results

### Background characteristics

The background characteristics of the trainees are shown in Table 1 . The median age of the trainees was 31 years (range, 27–43) and the median period of endoscopic training was 37 months (range, 4–102). Regarding the clinical experience of trainees, only 40% had experience with endoscopic hemostasis for emergency endoscopic treatments > 50 times, and 60% had experience with EMR or polypectomy of scheduled endoscopic treatment > 50 times.

**Table:** **Table 1** Background characteristics and experience of endoscopic treatment.

Gastroenterology residents (n = 50)				
Japanese: Canadian	38:12			
Age (years, median)	31 (27–43)			
Sex (male/female)	36/14			
Post graduate year (years, median)	6 (2–10)			
Length of training as a gastroenterology resident (months, median)	37 (4–102)			
Experience of endoscopic treatments (cases)	0–10	11–50	51–100	> 100
Endoscopic hemostasis	14%	46%	28%	12%
EMR/polypectomy	24%	16%	26%	34%
ESD	46%	44%	8%	2%
ERCP	40%	20%	18%	22%
EMR, endoscopic mucosal resection; ESD, endoscopic submucosal dissection; ERCP, endoscopic retrograde cholangiopancreatography.				

### Success rate and procedure time of endoscopic hemostasis with hemoclips

**Fig. 3:**
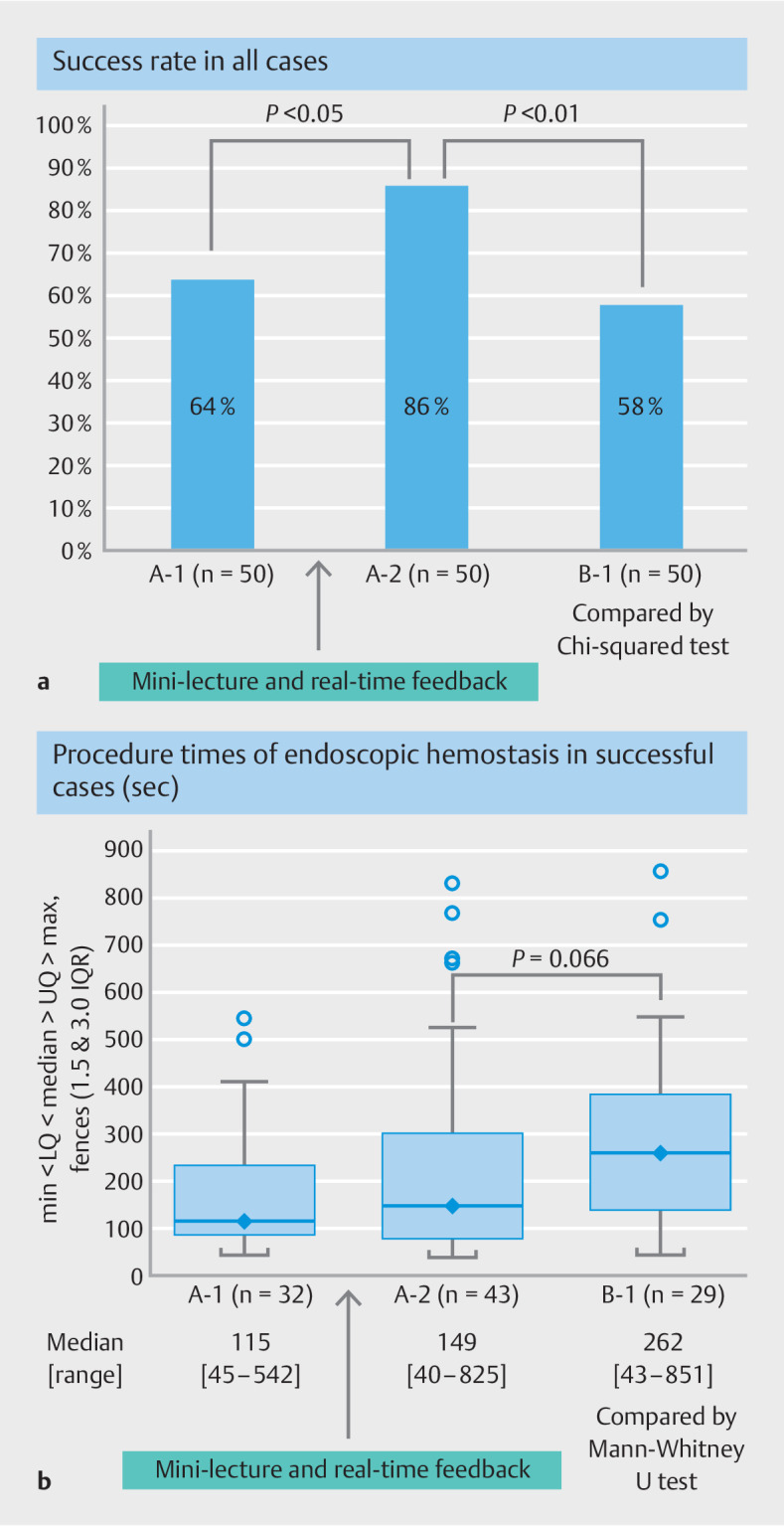
Success rate in **a** all cases and **b** procedure times of endoscopic hemostasis in successful cases. Median (♦): the mid-point of the data and the line dividing the box into two parts. The box represents the middle 50% of values for the group. 75% of the scores fell below the upper quartile. 25% of scores fell below the lower quartile. Outlier (○); LQ: lower quadrant; UQ: upper quadrant; IQR: interquartile range.

In a total of 50 residents, the hemostasis success rate for antral ulcer bleeding was significantly improved after instruction (64% for A-1 vs. 86% for A-2, *P* < 0.05). Meanwhile, the hemostasis success rate for ulcers in the upper body of the stomach was 58%, which was significantly lower than that for antral ulcers after instruction (86% for A-2 vs. 58% for B-1, *P* < 0.01) ( Fig. 3 **a** ). In Fig. 3 **b** , we compared the procedure times for successful cases after excluding the failed cases. The median procedure times before and after instruction did not show a significant difference (115 seconds for A-1 [range, 45–542 seconds] and 149 seconds for A-2 [range, 40–825 seconds], *P* = 0.56); however, compared with the procedure times for antral ulcers after instruction, ulcers in the upper body of the stomach tended to take more time (median procedure times: 149 seconds for A-2 and 262 seconds for B-1 [range, 43–851 seconds], *P* = 0.066).

We conducted a subanalysis to examine potential differences in success rate between the two countries, and between background experiences of each endoscopic procedure. (Supplementary Table 1). The subanalysis generally showed patterns consistent with the main analysis, indicating that A-2 had a better success rate than A-1, and B-1 had a lower success rate than A-2. Trainees with more than 50 endoscopic submucosal dissection cases achieved a higher hemostasis rate in both A-1 and B-1. However, this group was small with only five individuals, so the size limitation suggests being cautious about over-interpreting these findings.

### Comparison of trainee subjective assessment before and after simulator training

**Fig. 4:**
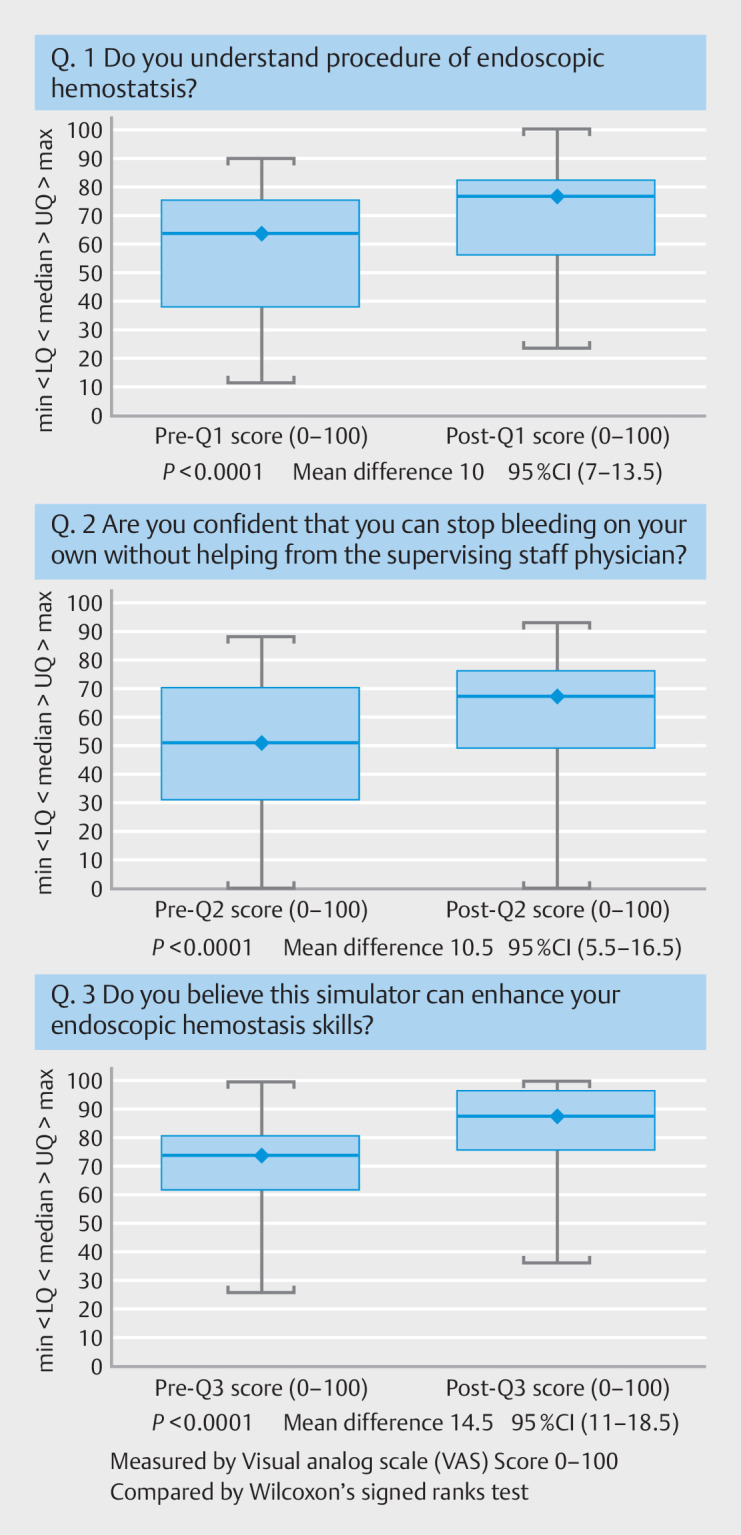
Comparison of trainee subjective assessment before and after simulation training. Median (♦): the mid-point of the data and the line dividing the box into two parts. The box represents the middle 50% of values for the group. 75% of the scores fell below the upper quartile. 25% of scores fell below the lower quartile. VAS, visual analog scale; CI: confidence interval, LQ: lower quadrant; UQ: upper quadrant; IQR: interquartile range.

To evaluate trainee understanding of the hemostatic procedure, confidence in performing the procedure without an instructor, and perceived effectiveness of model-based learning, we asked the following three questions: Q1: “Do you understand the procedure of endoscopic hemostasis?”; Q2: “Are you confident that you can perform endoscopic hemostasis on your own without the help of a supervisor?”; and Q3: “Do you believe this simulator can enhance your endoscopic hemostasis skills?” All VAS scores showed significant improvements after simulation training compared with those before training. The mean differences and 95% confidence intervals (CIs) for each question were 10 (95% CI: 7.0–13.5) for Q1, 10.5 (95% CI: 5.5–16.5) for Q2, and 14.5 (95% CI: 11.0–18.5) for Q3 ( Fig. 4 ).

### Text-mining by the co-occurrence network

**Fig. 5:**
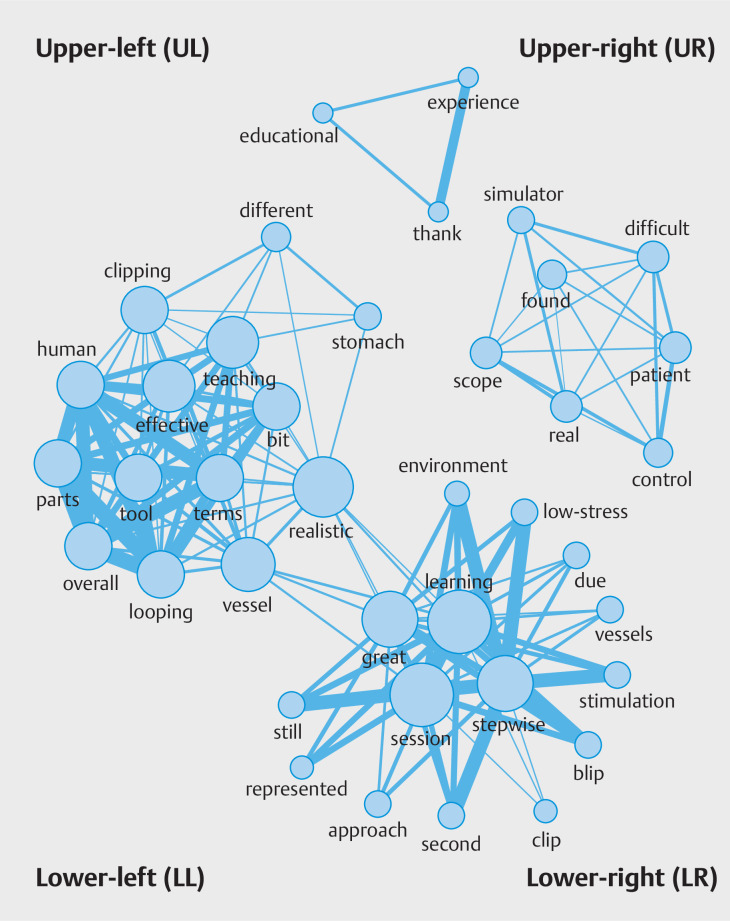
Text-mining using the co-occurrence network with a Jaccard index > 0.1. The Jaccard coefficient is the percentage of common elements between the elements in the two sets, and the coefficient is a number between 0 and 1. The connections between two words with a Jaccard index of 0.1 or higher are represented as lines, and the thicker the lines, the more frequently both words occurred simultaneously in trainee free comments. Source: Image courtesy of Mr. Yotaro Matsui and Prof. Manabu Ichikawa.

The results of text-mining are shown in Fig. 5 . There were four groups in the figure, and it could be confirmed that favorable words related to the simulation model co-occurred in most of them. We focused on three major groups. First, in the lower-right set, words related to the learning programs such as “learning,” “session,” and “stepwise” co-occurred with positive words such as “great” and “low-stress” in evaluations. Second, in the upper-left set, similarities and differences between the simulation model and the actual human body were mentioned for endoscopic hemostasis procedures performed in the stomach. Finally, the set in the upper-right corner represented the difficulty of controlling an endoscope in actual patients, as well as in the simulation training.

## Discussion

Herein, we evaluated the effectiveness of SBT for endoscopic hemostasis using hemoclips, which trainees may not encounter frequently in clinical practice. Our results suggest that this type of training is effective, particularly for trainees who do not have experience with a large number of endoscopic hemostasis cases owing to the procedure’s status as an emergency treatment. The proportion of trainees with experience with > 50 cases of endoscopic hemostasis was less than the proportion of those with experience with > 50 cases of EMR/polypectomy, as the latter is performed as a scheduled treatment. In addition, understanding and self-confidence regarding hemostatic techniques were low before training; these factors may be affected by the location and difficulty of hemostasis, which vary depending on the gastrointestinal bleeding cases that residents have experienced and not just the number of procedures performed. In addition, a survey of surgeons performing endoscopy in rural hospitals in North America found that 73% of them required training, particularly SBT with verbal feedback from expert endoscopists, for endoscopic management of nonvariceal upper gastrointestinal bleeding 19 . We found that by providing feedback and clearly stating specific learning objectives (three tips) during low-difficulty training, the success rate for the procedure increased significantly. This finding demonstrates that learning using SBT is beneficial for improving technical skills. Cohen et al. have reported that improving residents’ central venous catheter skills using SBT could reduce the incidence of catheter-related infections in intensive care units, reducing costs by > $700,000 per year 20 . The rebleeding rate after the initial endoscopic hemostasis for bleeding peptic ulcers was reported to be approximately 10% 21 22 . Furthermore, a randomized controlled trial (RCT) of peptic ulcer bleeding (the STING trial) showed that frequent recurrent bleeding after initial standard endoscopic hemostasis increased the cost of treatment compared with the cost of over-the-scope clips 23 . Therefore, improving endoscopic hemostatic techniques through effective SBT can help reduce treatment-related costs by lowering rebleeding rates.

When performing the procedure on the simulator at high-difficulty sites, the success rate was significantly lower compared with that of the second attempt at low-difficulty sites. This suggests that the model has a high reproducibility of difficulty, indicating the potential for its use as a learning tool, even for experienced practitioners to refine their skills. A comparison of treatment times within successful cases showed no significant reduction in treatment time before and after instruction. After instruction, treatment time tended to be longer for the more difficult sites than for the less difficult sites. Further, outliers were observed in all three groups, with a wide range of results from 40 seconds to >500 seconds. The tendency for longer treatment times at the difficult sites may indicate the difficulty in achieving a stable endoscopic visual field. However, the large overall variation in treatment time may have been influenced by the personalities of the learners or the fact that the learners took their time with the treatment because SBT is a learning method that does not involve sudden changes in patients such as hypovolemic shock.

There have been reports from a variety of fields that SBT reduces anxiety in clinical practice 24 25 . VAS evaluations before and after SBT showed significant increases in self-perceived proficiency, confidence in performing the procedure without an instructor, and perceived effectiveness of model-based learning. These findings indicate that SBT with this model contributes not only to learners’ skills but also to their confidence, suggesting superior outcomes compared with traditional educational methods based on on-the-job training.

Considering the co-occurrence network analysis of free comments after training, we inferred that a structured learning approach, as evidenced by the lower-right set, may contribute to a more effective and less stressful learning experience. The upper-left set highlights the importance of acknowledging both the strengths and limitations of the simulator model’s realism compared with real-life patient scenarios. Finally, the upper-right set suggests that continuous skill refinement is critical, given the challenges presented in controlling endoscopes during simulation training and in actual patient encounters. These additional insights further underscore the value of the novel endoscopic simulator as a training tool, while also recognizing areas for improvement and ongoing learning.

Because simulation-based medical education with deliberate practice is reported to be an effective learning process 26 , it is desirable to evaluate the long-term learning effects and the application of this training to actual clinical practice.

### Limitations

One limitation of our study is that we did not attempt to directly measure hemostasis success rates in real patients. However, due to the variable difficulty of endoscopic hemostatic procedures in emergency cases, it is challenging to investigate the hemostasis success rates in real patients as a true outcome. Recognizing this, we chose to compare success rates within our simulation model, which replicates the difficulty associated with bleeding sites. Therefore, we believe that the results of our study provide meaningful insights. We acknowledge that the sample size was relatively small, leading to limited generalizability of the findings to larger populations. In addition, the study did not evaluate the long-term retention of skills or their application in actual clinical practice. Furthermore, only two types of bleeding sites were included, which may not fully reflect the wide range of bleeding sites and severities encountered in real patients. In the clinical situation, endoscopists need to select an appropriate hemostatic method, not only clipping hemostasis but also factors such as coagulation grasper, injection, banding, and topical agents. This study for the simulator training did not include anything other than clipping. Finally, as a single-arm study, there is a potential for bias in the assessment of performance and subjective evaluations of trainees. A RCT with a non-intervention group based on a blinded third-party video evaluation of the procedure may be warranted.

## Conclusions

Our study demonstrated that the novel simulator learning model can be a valuable tool for improving the technical skills and confidence of trainees in performing endoscopic hemostasis.

## Supplementary Material

Supplementary material Zusatzmaterial
