# Supplementary material for: Interactive training with a novel simulation model for upper gastrointestinal endoscopic hemostasis improves trainee technique and confidence
Source: Endosc Int Open. 2024 Feb 28;12(2):E245–52. doi: 10.1055/a-2248-5110 (PMC10901645; doi:10.1055/a-2248-5110)
Supplement: Supplementary file 2 — Supplementary material [file 10-1055-a-2248-5110_22501017.pdf.pdf]

Supplementary material

Supplementary Table 1 Success rates between countries and experience with each endoscopic procedure.

		Success rate		
		**A-1**	**A-2**	**B-1**
**Trainee country**	**Canada (n = 12)**	**75% (9/12)**	**83% (10/12)**	**50% (6/12)**
	**Japan (n = 38)**	**61% (23/38)**	**87% (33/38)**	**61% (23/38)**
Experience with endoscopic treatments				
Endoscopic hemostasis	≤ 50 (n = 30)	67% (20/30)	83% (25/30)	60% (18/30)
	> 50 (n = 20)	60% (12/20)	90% (18/20)	55% (11/20)
EMR/polypectomy	≤ 50 (n = 20)	55% (11/20)	80% (16/20)	50% (10/20)
	> 50 (n = 30)	70% (21/30)	90% (27/30)	63% (19/30)
ESD	≤ 50 (n = 45)	62% (28/45)	89% (40/45)	56% (25/45)
	> 50 (n = 5)	80% (4/5)	60% (3/5)	80% (4/5)
ERCP	≤ 50 (n = 30)	67% (20/30)	83% (25/30)	57% (17/30)
	> 50 (n = 20)	60% (12/20)	90% (18/20)	60% (12/20)

EMR, endoscopic mucosal resection; ESD, endoscopic submucosal dissection; ERCP, endoscopic retrograde cholangiopancreatography.

A-1 is hemostasis for the antral ulcer bleeding before instruction.

A-2 is hemostasis for the antral ulcer bleeding after instruction.

B-1 is hemostasis for the ulcer in the posterior wall of the upper body of the stomach, following the A-2 hemostasis.

Supplementary material

Supplementary Fig. 1 The questionnaire form before the simulation training program included questions regarding background information and subjective assessments.

Entry Number # Facility ( ) Date (YYYY/MM/DD) / /

1. **Before using GI bleeding simulator**

・Sex (M / F)

・Age (years old)

・Current Year of training (PGY- _____)

・#Months of endoscopy training in actual patients so far (months)

・How many endoscopic procedures did you experience as an operator??

Please check ✓ the nearest number in box ☐ :Upper and/ or Lower GI

A) Endoscopic hemostasis (Clip, HSE, etc)

☐0-10 ☐11-50 ☐51-100 ☐Over 100

B) EMR (Endoscopic Mucosal Resection) and/ or Polypectomy

☐0-10 ☐11-50 ☐51-100 ☐Over 100

C) ESD (Endoscopic Submucosal Dissection)

☐0-10 ☐11-50 ☐51-100 ☐Over 100

D) ERCP (Endoscopic Retrograde Cholangio-Pancreatography)

☐0-10 ☐11-50 ☐51-100 ☐Over 100

Please answer the questions.

Example of answer
・Are you hungry？
Not at all ——X—— Extremely hungry

Q. 1 Do you understand procedure of endoscopic hemostasis?

Not at all |———| Excellent

Q. 2 Are you confident that you can stop bleeding on your own without helping from the supervising staff physician?

Strongly disagree |———| Strongly agree

Q. 3 Do you believe this simulator can enhance your endoscopic hemostasis skills?

Not at all |———| Excellent

Pre-questionnaire_

Supplementary material

Supplementary Figure 2 The questionnaire form after the simulation training program included records for the procedure, subjective assessments, and free comments.

Entry Number # Facility ( ) Date (YYYY/MM/DD) / /

2. After finished training with the simulator (Record by investigator)

・Procedure time to hemostasis (min:AntG-1, Success (clip(s)/ Failure)

(min:AntG-2, Success (clip(s)/ Failure)

(min:Body-1, Success (clip(s)/ Failure)

(min: Body-2, Success (clip(s)/ Failure)

※Definition: Time between starting bleeding and accomplish hemostasis or Time the trainee could not stop bleeding even had used 3 clips for one vessel (Up to 15 min for each ulcer model)

Please answer the questions. (From here **Answer by trainee themselves**)

Example of answer
・ Are you hungry？
Not at all ... Extremely hungry

Q. 1 Do you understand procedure of endoscopic hemostasis?

Not at all ... Excellent

Q. 2 Are you confident that you can stop bleeding on your own without helping from the supervising staff physician?

Strongly disagree ... Strongly agree

Post-questionnaire

Q. 3 Do you believe this simulator can improve your endoscopic hemostasis technique?

Not at all ... Excellent

Q. 4 Was this simulator an accurate depiction of bleeding in a real patient?

Not at all ... Excellent

Q. 5 Would you recommend this simulator to your colleagues or juniors?

Strongly disagree ... Strongly agree

Q. 6 Please write comments freely

(e.g. Differences between this simulator and actual patient care).
